# A Prospective Study on Challenges in the Management of Indeterminate Thyroid Nodules Without Molecular Testing

**DOI:** 10.1155/ije/4705557

**Published:** 2026-10-28

**Authors:** Jeyashanth Riju, Nihal Thomas, V. Thomas Paul, Deepak Thomas Abraham, M. Paul Jacob, J. Anne Prabhu, Marie Therese, Remya Rajan, J. Anish Cherian, Shawn Thomas, Rekha Pai, C. Rajiv Michael, J. Amit Tirkey, Natarajan Ramlingam, Elanthenral Sigamani, H. S. Asha, Nitin Kapoor, Felix Jebasingh, M. Gnanasekaran, A. Reetu John, P. G. Maya

**Affiliations:** ^1^ Department of Head and Neck Surgery Christian Medical College Vellore 632004 India cmch-vellore.edu; ^2^ Department of Endocrinology Diabetes and Metabolism Christian Medical College Vellore Centre for Stem Cell Research Christian Medical College Vellore Tamil Nadu India cmch-vellore.edu; ^3^ Department of Endocrinology Diabetes and Metabolism Christian Medical College Vellore 632004 India cmch-vellore.edu; ^4^ King Hamad American Mission Hospital A’ali Bahrain; ^5^ Department of Endocrine Surgery Christian Medical College Vellore 632004 India cmch-vellore.edu; ^6^ Department of Pathology Christian Medical College Vellore 632004 India cmch-vellore.edu; ^7^ Department of Radiodiagnosis Christian Medical College Vellore 632004 India cmch-vellore.edu; ^8^ Department of Medical Biostatistics Christian Medical College Vellore 632004 India cmch-vellore.edu

**Keywords:** cytodiagnosis, image-guided biopsy, interventional, thyroid nodule

## Abstract

**Introduction:**

Treating indeterminate thyroid nodules (ITNs) without validated molecular testing (MT) has been challenging. However, adding specific clinical and radiological parameters helps us to decide on treatment for a few individuals. The risk of malignancy in ITN after eliminating the obvious risk parameters needs to be studied.

**Methods:**

This prospective single‐centre analysis included patients who underwent fine needle aspiration cytology (FNAC) of thyroid between March 2022 and October 2023. The study included patients with thyroid nodules classified as Bethesda III/IV who subsequently underwent surgery. Different clinicopathological and FNAC‐related parameters were statistically analysed to predict malignancy, after excluding those factors that would strongly suggest malignancy.

**Results:**

Among 1501 patients who underwent FNAC, 207 met the inclusion criteria. The mean age of the study population was 39 ± 12.1 years, and the majority were women (80.2%). Among the ITNs, 86% were Bethesda III, and 14% were Bethesda IV nodules. The risk of malignancy in Bethesda III and IV nodules was 67.4% and 69%, respectively. On multivariate analysis, it was noted that hypothyroidism was the single independent factor that predicted malignancy associated with a significant odds of 0.309 (95%CI:0.127–0.784, *p* = 0.013). The most common malignancy among ITNs was the follicular variant of papillary thyroid carcinoma.

**Conclusion:**

Women less than 40 years of age, in this cohort, had a higher risk of malignancy compared to the Western population. More than half of the patients diagnosed with ITN underwent surgery. Hypothyroidism is an independent factor that may predict the risk of malignancy in ITNs, and this needs external validation. Rule‐in MT might be important in categorizing patients with ITN in the Indian population.

## 1. Introduction

Thyroid cancer is the second most common malignancy in Indian women aged 15–39 years [1]. A study done in the southern part of Kerala demonstrated an increased risk of thyroid cancer–related diagnoses in women less than 40 years of age; the concern of contracting cancer and the availability of several imaging diagnostic modalities had increased the number of thyroid cancer and surgeries [2].

Although The Bethesda System for Reporting Thyroid Cytopathology (TBSRTC) includes Bethesda III (atypia of indeterminate significance), Bethesda IV (Follicular neoplasm), and Bethesda V (suspicious of malignancy) as indeterminate cytopathologic interpretations, the management of Bethesda V nodules, which have a high risk of malignancy (67%–83%), remains surgery. In similar accords, the risk of malignancy in Bethesda III (13%–30%) or Bethesda IV (23%–34%) is lower, necessitating a different treatment strategy. The dilemma of performing surgery or follow‐up of patients has pertained to Bethesda III/IV category thyroid nodules [3]. Hence, these Bethesda III//IV–related nodules may be considered indeterminate thyroid nodules (ITNs) [4]. Auxiliary molecular testing (MT) may play a pivotal role and has been included in the routine management of ITNs. Although MT is validated and used regularly in diagnostic and treatment‐related planning in the Western population, the use of similar techniques in the European and Asian populations has been debated [5, 6]. At present, no validated form of MT is available in India [7].

Therefore, this study was designed to look into various preoperative factors that would predict malignancy in a nodule classified as ITN after eliminating all clinical and radiological features that would strongly suggest malignancy.

## 2. Materials and Methods

This prospective analysis includes patients who underwent thyroidectomy for ITNs in a tertiary care hospital between March 2022 and October 2023 in the Department of Head and Neck Surgery and Endocrine Surgery. This manuscript adheres to the STROBE guidelines for reporting the study (Supplement).

All patients who presented with thyroid swelling were screened clinically and radiologically, followed by ultrasound‐guided (USG) fine needle aspiration cytology (FNAC) from the dominant thyroid nodule. USG‐FNAC was done using a 6–13‐MHz probe of portable USG SonoSite MicroMAXX (USA) by the surgeon. FNAC was performed using a 24G needle with a nonsuction technique. Two or three needle passes were made, following which the adequacy of the material was checked by on‐field microscopy by a cytotechnician. The adequacy of FNAC was defined as the presence of a minimum of six groups of well‐visualized follicular cells. Each group should have a minimum of ten well‐preserved epithelial cells. In case of adequacy issues, especially bloody tap, clots were collected, the cell block was made for further evaluation and in the case of cystic lesions, the aspiration of the cyst was performed and the fluid was centrifuged and further evaluated. The FNAC outcomes were categorized into 6 groups based on TBSRTC [3]. The cytology was independently reviewed by two pathologists (AJP and MT) before the sample was labelled as ITN. The aim of the study was to analyse the factors predicting malignancy in ITNs, after excluding obvious clinic‐radiological risk factors that could suggest malignancy. The secondary objectives was to assess the histopathological characteristics and postoperative events following surgery in ITNs.

Patients over 18 years of age with proven ITN with the size of a thyroid nodule on clinical/radiological examination over 1 cm who were willing to undergo thyroid surgery were enrolled into the study. Need for thyroidectomy was based on surgeon discretion and patient logistics. Bethesda III//IV nodules were categorized as ITN. Further screening was done as mentioned in Figure 1, and patients who satisfied the inclusion and exclusion criteria were included in the study. Comprehensive counselling was given depending on the clinical and radiological features. The disease management and its prognosis were explained. Written informed consent was obtained prior to the procedure. Preoperative procedures included routine blood investigations and preoperative vocal cord assessment.

**FIGURE 1:**
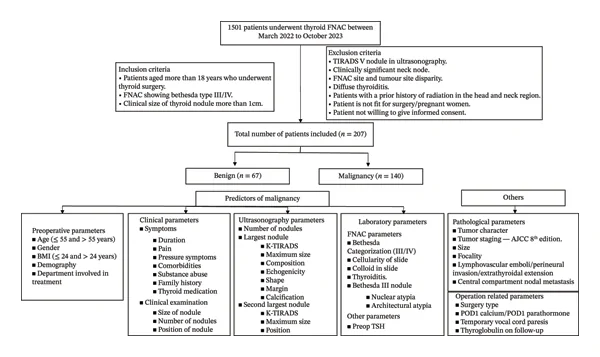
Study flowchart.

A number of clinical, radiological, laboratory, pathological, and operation‐related parameters, as mentioned in Figure 1, were analysed. The thyroid nodule was radiologically classified based on K‐TIRADS, and radiological parameters were considered [8, 9]. Carcinomas measuring ≤ 1.0 cm were termed microcarcinoma [10].

The type of surgery and various postsurgical morbidities of surgery were documented. Transient or temporary hypoparathyroidism was defined as a postoperative PTH level of less than 10 pg/mL. Transient or temporary hypocalcaemia was defined as a postoperative serum calcium level of less than 8 mg/mL [11]. The vocal cord paresis rate following surgery was also recorded. Hypothyroidism was defined as the use of thyroid hormone replacement during or before surgery, and all patients were euthyroid during surgery [12].

The data were entered and analysed using SPSS 21.0 software The categorical variables were expressed as frequencies and percentages. The continuous variables were summarized using mean and standard deviation. Regarding the interquartile range (IQR), the upper and lower quartiles were expressed as the 75th and 25th percentiles, respectively. The association between categorical variables were assessed using Fisher’s exact test. Univariate and multivariate analyses for preoperative factors predicting malignancy were done using logistic regression. A *p* value ≤ 0.05 was considered to be statistically significant. Spearman’s rank correlation was used to assess the strength of association between variables.

## 3. Results

The study included a total of 1501 FNACs, and among them, 486 (32.4%) were classified as ITN. Among the patients with ITN, 250 underwent surgery (51.3%). In the group who had surgery, 30 patients with TIRADS 5 nodules (83.3% had malignancy) and 10 patients with FNAC and tumour site disparity and three patients without baseline ultrasound done in our centre were excluded. A total of 207 patients with FNAC‐classified ITN were included in the final analysis.

The mean age of the study population was 39 ± 12.1 years (range: 18 to 77 years), and the majority were women (80.2%). 67.6% (140 patients) had malignancy in the final histopathological examination (HPE) (Table 1); the risk would come down to 55.1% if those with microcarcinoma were excluded.

**TABLE 1:** Univariate analysis of preoperative baseline parameters predicting malignancy in indeterminate thyroid nodules.

Variables	Benign + NIFTP (*n* = 67) (32.4%)	Malignancy (*n* = 140) (67.6%)	Odds ratio (95% CI)	*p* Value
Age				
≤ 55 years	61 (91%)	122 (87.1%)	1.5 (0.567‐3.972)	0.492
> 55 years	6 (9%)	18 (12.9%)		
Gender				
Women	57 (34.3%)	109 (65.7%)	1.621 (0.742‐ 3.542)	0.226
Men	10 (24.4%)	31 (75.6%)		
RNA extracted				
≤ 6.5	24 (60%)	26 (44.8%)	1.846 (0.815‐4.180)	0.156
> 6.5	16 (40%)	32 (55.2%)		
BMI				
≤ 24kg/m^2^	35 (52.2%)	74 (52.9%)	0.976 (0.545‐1.748)	1.000
> 24kg/m^2^	32 (47.8%)	66 (47.1%)		
Demography				
South India	19 (28.4%)	24 (17.1%)	1.861 (0.911–3.803) 2.087 (0.833–5.230)	**0.088** 0.116
North India	37 (55.2%)	87 (62.1%)		
Others	11 (16.4%)	29 (20.7%)		

*Note:* Bold values indicate *p* value ≤ 0.10.

Three‐fourths of the patients over 55 years had malignancy; however, the age‐related criteria could not predict malignancy (*p* = 0.492).

Incidental detection of thyroid nodules was noted in 10 patients (4.8%). Among them, 8 patients had malignancy. Two of the subjects had a family history of thyroid malignancy and had follicular variant of papillary thyroid carcinoma (FvPTC) in the final histopathology report. The patients who were on hypothyroid medications on presentation had a lower risk of malignancy when compared to those who were not on medication, which was significant on univariate analysis (95% CI:0.339 [0.138–0.832], *p* = 0.018) (Table 2).

**TABLE 2:** Univariate analysis of preoperative clinical parameters predicting malignancy in indeterminate thyroid nodules.

Variables	Benign + NIFTP (*n* = 67) (32.4%)	Malignancy (*n* = 140) (67.6%)	Odds ratio (95% CI)	*p* value
Duration of symptoms				
≤ 12 months	38 (56.7%)	70 (50%)	1.310 (0.729‐2.354)	0.377
> 12 months	29 (43.3%)	70 (50%)		
Pain as symptom				
No	64 (95.5%)	132 (94.3%)	1.123 (0.281–4.485)	0.870
Yes	3 (4.5%)	7 (5.0%)		
Pressure symptoms				
No	63 (94%)	123 (87.9%)	2.177 (0.703–6.744)	0.178
Yes	4 (6%)	17 (12.1%)		
Comorbidities				
No	48 (71.6%)	106 (75.7%)	0.810 (0.420–1.563)	0.530
Yes	19 (28.4%)	34 (24.3%)		
Tobacco/alcohol habits				
No	62 (92.5%)	127 (90.7%)	1.269 (0.433–3.720)	0.664
Yes	5 (7.5%)	13 (9.3%)		
Family history of malignancy				
No	67 (100%)	138 (98.6%)	1.486 (1.350–1.634)	0.999
Yes	0 (0%)	2 (1.4%)		
Thyroid‐related medication				
No	52 (77.6%)	128 (91.4%)	0.339 (0.138–0.832) 0.271 (0.044–1.668)	**0.018** 0.159
Hypothyroid medication	12 (17.9%)	10 (7.1%)		
Hyperthyroid medication	3 (4.5%)	2 (1.4%)		
Clinical examination—nodule size				
≤ 4cm	47 (70.1%)	89 (63.6%)	1.347 (0.720‐2.519)	0.434
> 4cm	20 (29.9%)	51 (36.4%)		
Clinical examination—nodule number				
Solitary	55 (82.1%)	119 (85%)	0.578 (0.149–2.235) 0.924 (0.373–2.289)	0.427 0.865
Multinodular goitre	4 (6%)	5 (3.6%)		
Multinodular goitre with dominant nodule	8 (11.9%)	16 (11.4%)		
Clinical examination of the right lobe				
No palpable nodule	19 (28.4%)	47 ( 33.6%)	0.783 (0.414–1.480)	0.525
Palpable nodule	48 (71.6%)	93 (66.4%)		
Clinical examination of the left lobe				
No palpable nodule	43 (64.2%)	79 (56.4%)	1.383 (0.759–2.523)	0.365
Palpable nodule	24 (35.8%)	61 (43.6%)		
Clinical examination of isthmus				
No palpable nodule	58 (86.6%)	125 (89.3%)	0.773 (0.320–1.870)	0.644
Palpable nodule	9 (13.4%)	15 (10.7%)		

*Note:* Bold values indicate *p* value ≤ 0.10.

The median size of the dominant thyroid nodule on clinical examination was 4 cm (range 1 to 15 cm) and the majority (84.1%) presented as a solitary nodular goitre. 72.9% of the nodules that were more than 4 cm on clinical examination were malignant compared to 65.4% in the counterpart, but this did not predict malignancy (*p* = 0.434). Neither the number of nodules on examination nor the site of the nodules on clinical examination predicted malignancy.

The median size of the thyroid nodule on USG was 3.5 cm (range 1 cm to 15.7 cm), and solitary nodular goitre was noted in 48.3% of the individuals. Ultrasound findings such as classification based on K‐TIRADS, size of the nodule (*p* = 0.360), number of nodules (*p* = 1), nodule composition (*p* = 0.454), echogenicity, the shape of the nodule (*p* = 1), irregularity of margin (*p* = 0.730) and calcification (*p* = 0.359) were not able to predict malignancy due to strict inclusion. USG evaluation of the second largest nodule based on K‐TIRADS (*p* = 0.801), size (*p* = 0.822) and position (0.839) had no significant influence on predicting malignancy (Table 3).

**TABLE 3:** Univariate analysis of preoperative investigation–related parameters predicting malignancy in indeterminate thyroid nodules.

Variables	Benign + NIFTP (*n* = 67) (32.4%)	Malignancy (*n* = 140) (67.6%)	Odds ratio (95% CI)	*p* value
K‐TIRADS				
2	3 (4.5%)	10 (7.1%)	0.615 (0.160–2.35) 0.60 (0.151–2.385)	0.479 0.468
3	40 (59.7%)	82 (58.6%)		
4	24 (35.8%)	48 (34.3%)		
USG evaluation—maximum size of the nodule				
≤ 40mm	39 (58.2%)	91 (65%)	0.750 (0.413–1.362)	0.360
> 40mm	28 (41.8%)	49 (35%)		
USG evaluation—number of thyroid nodules				
Single nodule	32 (47.8%)	68 (48.6%)	0.968 (0.541–1.734)	1.000
Multiple nodules	35 (52.2%)	72 (51.4%)		
USG evaluation—nodule component				
Solid	35 (52.2%)	82 (58.6%)	0.774 (0.431–1.389)	0.454
Mixed	32 (47.8%)	58 (41.4%)		
USG evaluation—nodule echogenicity				
Hyperechoic	26 (38.8%)	47 (33.6%)	0.593 (0.248–1.417) 1.598 (0.835–3.058)	0.240 0.157
Hypoechoic	14 (20.9%)	15 (10.7%)		
Isoechoic	27 (40.3%)	78 (55.7%)		
USG evaluation—shape of the nodule				
Wider than taller	67 (100%)	138 (98.6%)	0.673 (0.612–0.741)	1.000
Taller than wider	0 (0%)	2 (1.4%)		
USG evaluation—margin of the nodule				
Smooth	63 (94%)	134 (95.7%)	0.705 (0.192–2.588)	0.730
Irregular	4 (6%)	6 (4.3%)		
USG evaluation—calcification				
No	53 (79.1%)	118 (84.3%)	0.706 (0.335–1.493)	0.359
Yes	14 (20.9%)	22 (15.7%)		
USG evaluation—size of the next dominant nodule				
≤ 10mm	10 (37%)	31 (40.3%)	0.873 (0.353–2.156)	0.822
> 10mm	17 (63%)	46 (59.7%)		
USG evaluation—K‐TIRADS of the next dominant nodule				
TIRADS ≤ 3	22 (81.5%)	61 (79.2%)	1.154 (0.378–3.524)	0.801
TIRADS > 3	5 (18.5%)	16 (20.8%)		
USG evaluation—position of the next dominant nodule				
Ipsilateral	9 (34.6%)	28 (36.8%)	0.908 (0.357–2.307)	0.839
Contralateral	17 (65.4%)	48 (63.2%)		
USG evaluation—presence or absence of the nodule in the contralateral lobe				
No	44 (65.7%)	82 (58.6%)	1.353 (0.738–2.481)	0.363
Yes	23 (34.3%)	58 (41.4%)		
FNAC characteristics—Bethesda categorization				
III	58 (86.6%)	120 (85.7%)	1.074 (0.461–2.505)	0.869
IV	9 (13.4%)	20 (14.3%)		
FNAC characteristics—nuclear atypia				
No	16 (36.4%)	25 (24.5%)	1.76 (0.821–3.771)	0.146
Yes	28 (63.6%)	77 (75.5%)		
FNAC characteristics—architectural atypia				
No	10 (22.7%)	37 (36.3%)	0.517 (0.229–1.164)	0.111
Yes	34 (77.3%)	65 (63.7%)		
FNAC characteristics—nuclear atypia and architectural atypia combined				
No	26 (59.1%)	63 (61.8%)	0.894 (0.435–1.840)	0.761
Yes	18 (40.9%)	39 (38.2%)		
FNAC characteristics—cellularity of slide				
Scanty	39 (60%)	85 (63%)	0.847 (0.460–1.558) Exp	0.594 0.999
Adequate	26 (40%)	48 (35.6%)		
Nil	0	2 (1.5%)		
FNAC characteristics—colloid of slide				
Scanty	36 (57.1%)	85 (66.4%)	0.715 (0.344–1.484) 0.616 (0.260–1.457)	0.368 0.270
Adequate	16 (25.4%)	27 (21.1%)		
Nil	11 (17.5%)	16 (12.5%)		
FNAC characteristics—thryoiditis				
No	49 (73.1%)	117 (83.6%)	0.535 (0.265–1.079)	**0.094**
Yes	18 (26.9%)	23 (16.4%)		
Preoperative TSH level				
**≤** 1.7 mIU/L	32 (47.8%)	75 (53.6%)	0.792 (0.442‐1.420)	0.434
> 1.7 mIU/L	35 (52.2%)	65 (46.4%)		
Preoperative fT4 level				
fT4 ≤ 1.2 ng/dL	26 (59.1%)	53 (67.1%)	0.709 (0.331–1.519)	0.376
fT4 > 1.2 ng/dL	18 (40.9%)	26 (32.9%)		

*Note:* Bold values indicate *p* value ≤ 0.10.

Among the patients who were classified to have Bethesda III nodules (86%), 67.4 had malignancy, while 69% of nodules who were classified as Bethesda IV had malignancy in the final HPE, with no statistical difference between these groups (*p* = 0.869) (Figure 2). Among FNACs that were classified as Bethesda III nodules, 73.3% with nuclear atypia and 65.7% with architectural atypia had malignancy on the final histopathology, but neither of these factors could independently predict malignancy. Examination of the slides showed an absence or scanty cellularity in 60.9% of subjects and nil or scanty colloid in 71.5%. Corresponding malignancy in the final histopathology was noted in 70.2% and 68.2% of subjects, respectively.

**FIGURE 2:**
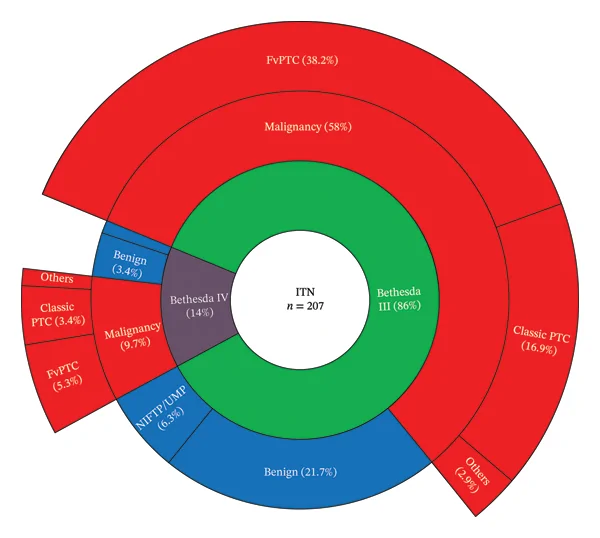
Sunburst chart illustrating the distribution of neoplasm under indeterminate thyroid nodules. *Note:* FvPTC‐Follicular variant of papillary thyroid carcinoma; NIFTP, noninvasive follicular thyroid neoplasm with papillary‐like nuclear features (abbreviation: UMP, uncertain malignant potential).

In the multivariate analysis (Table 4), patients on hypothyroid medication had a significantly lower odds of thyroid malignancy (69.1%; 95% CI [0.124–0.775], *p* = 0.012), controlling for other factors in the model.

**TABLE 4:** Multivariate analysis of significant univariate factors influencing malignancy in indeterminate thyroid nodules.

Parameters	*p* value	Odd’s ratio	95% confidence interval	
			Lower	Upper
Thyroid‐related medication				
Hypothyroid medication	**0.012**	0.309	0.124	0.775
Hyperthyroid medication	0.252	0.335	0.052	2.174
Demography				
North India	0.089	1.900	0.907	3.979
Others	0.127	2.083	0.811	5.352
FNAC characteristics				
Thyroiditis	0.111	0.553	0.267	1.145

*Note:* Bold values indicate *p* value ≤ 0.05.

The median size of tumour foci on microscopic examination was 30 mm (IQR: 17 mm–41 mm). The majority of the malignancies were FvPTC (64.3%), followed by classical PTC (30%), medullary thyroid carcinoma (2.9%), high‐grade differentiated thyroid carcinoma (2.1%) and tall cell variant of PTC (0.7%). Noninvasive follicular thyroid neoplasm with papillary‐like nuclear features (NIFTP) and well‐differentiated tumours of uncertain malignant potential (WT‐UMP) contributed to 7.2% of the histopathological diagnosis (15 of 207 specimens), of which 13/207 occurred in Bethesda III and the rest in Bethesda IV nodules (Figure 2).

Among those with malignancy, 28.6%, 40.7% and 30.7% belonged to AJCC eighth edition Tumour Stages 1, 2 and 3, respectively. Multifocality was observed in 64.3% of the malignant subjects, and 50% of the subjects had malignancy in the contralateral thyroid lobe. Clinical examination showed a solitary nodule in 119 of the malignancy subjects, while only 40.3% had a single focus. Although USG demonstrated a single nodule in 68 subjects, only 33.8% had single foci on final histopathology. The Spearman’s correlation on the number of tumour foci in patients with malignant nodules showed no significant correlation between clinical examination and USG finding (*r* = 0.042, *n* = 140, *p* = 0.624), while a weak but significant correlation was noted between clinical examination and HPE (*r* = 0.281, *n* = 140, *p* = 0.001). Lymphovascular emboli, perineural invasion and extrathyroidal extension on microscopy were noted in 22.9%, 1.4% and 3.5% of the subjects, respectively. Central compartment‐related nodal metastasis was noted in 6.4% of cases.

One hundred and fifty‐eight patients underwent total thyroidectomy (76.3%), of whom central compartment neck dissection was done in 6 patients (3%) based on the intraoperative findings. All those with significant central compartment neck nodes had malignancy. Malignancy was noted in 75.3% of patients who underwent total thyroidectomy. In patients who underwent hemithyroidectomy (23.7%), 39.5% had malignancy, and completion thyroidectomy was performed in 11 of the 15 malignant subjects.

One patient underwent emergency re‐exploration following surgery for hematoma. Following surgery, 13 patients had hypocalcaemia (7.6%), and 21.6% had hypoparathyroidism postsurgery (PTH ≤ 10 pg/mL) on postoperative Day 1. Two patients had to be readmitted for the treatment of hypocalcaemia. Of the 376 recurrent laryngeal nerves at risk during thyroid surgery, transient vocal cord palsy was noted in 3 patients (0.8%) and all three had malignancy on HPE. The median stimulated thyroglobulin value was 2.6 ng/mL (IQR = 0.40–5.63) during the first follow‐up period, postsurgery.

On comparing the characteristics of operated versus nonoperated patients in the entire cohort of ITN, patients who underwent surgery had significantly higher proportion of patients aged ≤ 55 years (aOR 2.92, 95% CI 1.79–4.77; *p* < 0.001), higher proportion of nonlocal residents (adjusted OR 1.75, 95% CI 1.11 – 2.70; *p* = 0.014), more Bethesda IV nodules (aOR 6.25, 95% CI 2.50–16.66: *p* < 0.001) and nodules > 40 mm (aOR 1.56 95% CI 1.04 – 2.32; *p* = 0.030) compared to patients kept under surveillance (Table 5).

**TABLE 5:** Predictors of surgical management among patients with Bethesda III/IV thyroid nodules: Univariate and multivariate logistic regression analysis.

Variables	Operated (*n* = 250) (51.3%)	Not operated (*n* = 246) (50.7%)	Unadjusted OR (95% CI)	Univariate *p* value	Adjusted OR (95% CI)	Multivariate *p* value
Age						
≤ 55 years	220 (88%)	166 (70%)	3.13 (1.96–5.03)	**<** **0.001**	2.92 (1.79–4.77)	**<** **0.001**
> 55 years	30 (12%)	71 (30%)				
Gender						
Male	55 (22%)	39 (16.5%)	1.43 (0.9–2.26)	0.122	—	—
Female	195 (78%)	198 (83.5%)				
Demography						
South India	49 (19.6%)	75 (31.6%)	1.89 (1.25–2.86)	**0.002**	1.75 (1.11–2.70)	**0.014**
Rest of India	201 (80.4%)	162 (68.4%)				
K‐TIRADS						
≤ 3	142 (56.8%)	135 (57%)	1.01 (0.70–1.45)	0.522	—	—
> 4	108 (43.2%)	102 (43%)				
USG evaluation—maximum size of the nodule						
≤ 40 mm	158 (63.2%)	169 (71.3%)	1.45 (0.99–2.13)	0.057	1.56 (1.04–2.33)	**0.030**
> 40 mm	92 (36.8%)	68 (28.7%)				
FNAC characteristics—Bethesda categorization						
III	218 (87.2%)	231 (97.5%)	5.56 (2.33–14.29)	**<** **0.001**	6.25 (2.50–16.67)	**<** **0.001**
IV	32 (12.8%)	6 (2.5%)				
TSH level						
≤ 1.7 mIU/L	122 (48.8%)	110 (46.4%)	1.1 (0.77–1.57)	0.331	—	—
> 1.7 mIU/L	128 (51.2%)	127 (53.6%)				

*Note:* Bold values indicate *p* value ≤ 0.05.

### 3.1. Discussion

Thyroid nodules are usually managed based on the results of clinical examination, FNAC and USG neck findings; FNAC is not indicated when the nodule size is less than 1 cm [13]. FNAC‐based planning is an important standard of care for treating thyroid nodules based on the standard guidelines [5]. TBSRTC has been most frequently used in the classification and management of thyroid nodules. However, when dealing with ITN, FNAC cannot be used as a stand‐alone criterion. A number of other tumour‐related clinical and radiological factors need to be considered while planning management in the absence of validated MT.

Studies have shown that TIRADS 5 nodules have a high risk of malignancy; a study from our institution noted that 97.4% of nodules classified as TIRADS 5 were malignant, so we excluded patients with TIRADS 5 thyroid nodules in this study [8, 9]. We noticed that of the 30 patients with TIRADS 5 nodules, 25 subjects had malignancy (83.3%), 2 had NIFTP (6.7%), and 3 were benign (10%) (OR.2.39: 95% CI [0.88–6.53], *p* = 0.088). Analysis of various parameters of TIRADS 5 nodules showed that most of the subjects with TIRADS 5 nodule has classic PTC compared to FvPTc and others (OR.2.73: 95% CI [1.14–6.51], *p* = 0.024), and TIRADS 5 nodules were relatively smaller compared to the others (25 mm  vs 35 mm OR.0.97: 95%CI [0.94–0.99], *p* = 0.003). Among the USG characteristics, the presence of calcification is an important character in nodules to be classified as TIRADS 5 (OR.13.05: 95% CI [5.39–31.66], *p* < 0.001) (supporting table S1). It is also known that an ITN nodule in the setting of distant metastatic disease or neck nodal metastasis is generally malignant. Therefore, surgery is mostly mandated in patients with these factors, which are strongly suggestive of malignancy and were therefore excluded.

In our study, we found a high prevalence of malignancy among the ITN subjects, at 67.6% (see Figure 2). This result is consistent with findings from other studies conducted in India. For instance, a retrospective study by Thadathil et al. reported a malignancy prevalence of 66.5% in a South Indian population. Similarly, Thiagarajan et al. reported a prevalence of 77.2% in patients from a tertiary care hospital in India [14, 15]. However, selection bias also plays a significant role. We noticed that patient selection was independently influenced by age, geographic location, Bethesda category and thyroid nodule size at presentation (Table 5). These findings suggest that older patients (> 55 years) choose surveillance, and a higher proportion from the local regions (South Indian) were under surveillance rather than surgery, as it is easier to follow up. The higher surgical rates observed among Bethesda IV nodules likely reflect their greater estimated risk of malignancy compared with Bethesda III nodules. Similarly, patients with nodules > 40 mm were more likely to undergo surgery, potentially owing to concerns regarding malignancy risk, compressive symptoms, cosmetic considerations or physician recommendation.

Similar to a meta‐analysis by Trimboli et al., our study also demonstrated a preponderance among women (80.2%) [4]. Studies had demonstrated a higher risk of malignancy among men; although the odds ratio was higher in our group (OR.1.621: 95% CI [0.742–3.542], *p* = 0.226), it did not meet statistical significance [4, 13]. Although multiple studies have noted the prevalence of malignancy to be higher in the elderly, similar findings were not noted in our study; on the contrary, we noted that the median age of our population who were diagnosed with malignancy was younger, 38.5 years (IQR: 30–48 years) [4, 5, 16]. Epidemiological studies in India have noted women less than 40 years of age have a higher magnitude of thyroid cancer; the same finding was reported in our study [1, 17].

Although the TBSRTC system has been followed in many countries, the number of patients falling into each category varies, and more so does the incidence of malignancy in each category. A meta‐analysis by Bongiovanni et al. reported that the prevalence of ITN can range up to 31.6%. We noted a higher prevalence of ITN in our population (32.4%). Also, the incidence of malignancy in ITN was over 50% in studies conducted in the Asian population; a similar result was demonstrated in our study [5, 18]. Such differences might be triggered by different geographical locations, referral systems and selection bias where the study was conducted. The median size of nodules on HPE was bigger (37 mm; IQR 24 to 45 mm) compared to the study by Angell et al. (2.2 cm) or Yip et al. (2.1 cm) [12, 16]. The bigger nodule size might be responsible for the higher prevalence of malignancy in ITN due to the limitation in the FNAC sampling area.

MT play an important role in the management of ITN. A molecular test is labelled as a ‘rule‐in’ when it exhibits high specificity and a high positive predictive value (PPV). A positive test result suggests a high likelihood of malignancy in the specimen, leading to surgery as the next step. When there is a strong suspicion of malignancy in a patient with an ITN, a rule‐in test is preferable, as in our cohort [19–21]. The minimal PPV of a test is intended to align closely with the 50%–75% range associated with the Bethesda V nodule classification. As a result, many studies have aimed for a PPV exceeding 60% [22]. With a higher prevalence of malignancy in our population, a ‘rule‐in’ test appears beneficial.

The risk of malignancy when the nodule size is more than 4 cm is debated [4, 16]. We noticed that 7 of 27 patients (25.9%) with microcarcinoma had gross thyroid nodules with a size greater than 4 cm on HPE (median 22 mm IQR:15 mm to 41 mm).

The majority of microcarcinoma were FvPTC (66.7%), and the rest were classic PTC (33.3%). Among the various histopathological parameters in microcarcinoma, extrathyroidal extension, perineural invasion, lymphovascular emboli, multifocality and central compartment nodal metastasis were noted in 3.7%, 3.7%, 14.8%, 55.6% and 11.1%, respectively. Of the 22 patients with microcarcinoma who underwent total thyroidectomy, contralateral lobe involvement was noted in 45.5% of subjects. The above factors, especially multifocality, could predict recurrence, indicating that microcarcinoma in ITN may need to be managed similarly to other PTCs, and the need for complete thyroid resection has to be discussed with the patient [10, 23].

Many had tried dividing Bethesda III nodules based on nuclear and architectural atypia to help the clinical team predict malignancy. It has been noted that nuclear atypia has a higher risk of malignancy than architectural atypia [24, 25]. We noticed the risk of malignancy was 73.3% for nodules with nuclear atypia, 65.7% for those with architectural atypia, and 68.4% for nodules with both nuclear atypia and architectural atypia. However, this was not statistically significant in predicting malignancy. Interestingly, among patients with NIFTP/UMP, 81.8% had architectural atypia, while only 27.3% had nuclear atypia.

Hypothyroidism has been noted to be linked with multiple malignancies, which include thyroid, breast, colon, prostate and lung [26, 27]. With regard to thyroid cancer, a higher level of TSH will stimulate thyroid follicular cells and has been thought to influence the progression of thyroid carcinoma to an advanced stage [27, 28]. We noticed patients on hypothyroid medication had significantly lower odds of having thyroid malignancy by approximately 69.1% (95% CI: 0.309 [0.124–0.775], *p* = 0.012), controlling for other factors in the model. This effect is statistically significant and suggests a strong protective effect of this medication against the outcome. All patients in this hypothyroid group were less than 55 years old, except one patient with a tumour foci of less than 1 cm (median 39 mm IQR 24 to 61 mm). Most of the patients in this group had FvPTC (40%), followed by classical PTC (30%). It may be postulated that a patient on treatment for hypothyroidism may have an initial higher TSH, which is responsible for the stimulation of thyroid follicular cells leading to goitre, and such patients may tend to get evaluated early and picked up for surgery since hypothyroidism is believed to be associated with cancer. Furthermore, an exogenous supply of thyroxine may reduce the TSH secretion and, in turn, reduce the thyroid stimulation or possible conversion to cancer [27, 29]. The observed association between hypothyroid medication use and lower malignancy risk is notable but should be interpreted cautiously and should be considered hypothesis‐generating rather than causal, because on further analysis, neither TSH nor free T4 showed a direct effect on malignancy or aggressive tumour biology (Supporting table S2). Hence, this needs to be externally validated in larger cohorts in multiple centres, with complete thyroid‐function profiles, thyroid antibody status, levothyroxine dose and duration, and longitudinal TSH control.

Most of the subjects (75.4%) in this study underwent total thyroidectomy. Selection of the type of surgery in an Indian scenario has many practical challenges, with many patients opting for total thyroidectomy, especially in the age group over 55 years (91.7%) [12, 13, 30]. The reasons are the possible need and fear of resurgery, financial/logistic issues that will compromise their daily work, ability to follow up, the possibility of multifocality if malignant (64.3% in the present study) and fear of cancer. Surgeon‐related factors that would favour a total thyroidectomy include more than 1‐cm‐sized nodules in the contralateral lobe, family history of thyroid malignancy, radiation exposure, fluorodeoxyglucose avidity on positron emission tomography scan, prior history of hypothyroidism and patients with multiple comorbidities [12].

Various meta‐analyses had demonstrated that the risk of postoperative complications like haemorrhage to be between 0.43% and 4.39%, the risk of recurrent laryngeal nerve injury rate ranged 0% to 5.9%, and the rate of transient hypocalcaemia was noted to be between 19% to 27% of subjects [31–33]. Compared to the meta‐analyses that were published, the risk was lower in our study group probably attributed to the experience of the surgeon.

The role of repeat FNAC has been recommended in treatment guidelines and multiple studies [3, 34]. A meta‐analysis by Ooi et al. of 7541 subjects with ITN in the Asian population found that repeat FNAC was performed in 29.4% of cases, resulting in a change in Bethesda categorization in 65.9% of cases. However, the same study also noted that performing a repeat FNAC did not have a statistically significant impact on the risk of malignancy after surgical resection, when compared to patients who underwent surgery without a repeat FNAC [35]. As the incidence of malignancy is higher in our population, the role of repeat FNAC may be different from other institutions and needs to be further investigated. Also, a repeat FNAC was not considered due to logistical constraints and the patient’s personal preference.

USG plays a crucial role in the preliminary management of ITN and in determining the need for FNAC. Once the FNAC results indicate ITN, further diagnostic evaluations such as MT, repeat FNAC and the potential need for surgery may be considered. Currently, to reduce unnecessary FNAC and to support further management, artificial intelligence (AI)–assisted decision‐making may be considered [36]. Thyroid nodule visualization (TNVis) uses dynamic ultrasound video and 3D visualization to improve junior radiologist performance, raising AUC from 0.59 without assistance to 0.79 with full assistance, while ThyGPT integrates multimodal imaging and language interaction, improving radiologists’ AUC from 0.805 to 0.908, reducing biopsy rates by over 40% and detecting ultrasound‐report errors far faster than humans [37, 38]. A study by Yao noted that the AI model can extend this logic to Bethesda IV nodules, where FNAC often cannot distinguish benign follicular lesions from follicular thyroid carcinoma or FvPTC; the ultrasound AI models achieved high AUCs around 0.90–0.95, suggesting that AI may help refine management of Bethesda IV nodules [39]. It appears that AI is currently unlikely to replace clinicians, but it can become a powerful clinical copilot for thyroid nodule diagnosis and management by improving consistency, interpretability, report quality and patient‐specific decision‐making; however, these tools still require careful validation across populations, ultrasound devices, institutions and rare tumour subtypes before they can be safely embedded into routine practice.

The strength of the current study is that we have a prospective data analysis after eliminating parameters that would directly suggest malignancy. The study has some limitations. These include it being a single‐institution–based study. Our centre is a tertiary care hospital; therefore, external referral bias may be of importance. Patient selection bias was present since we operated on individuals not solely based on FNAC but also based on clinical and radiological features. Although the utility of repeat FNAC has been included under Bethesda III nodule management, our patient group did not undergo repeat FNAC testing.

This work was previously presented as a poster at the 24th FHNO Conference 2024 and subsequently published as a conference abstract [40].

## 4. Conclusion

Thyroid malignancy affects the younger age group in the Indian population, and the size of nodules is comparatively bigger. The risk of malignancy in ITN is higher. Microcarcinoma occurring in ITN also has a greater chance for multifocality. After eliminating clinical and radiological risk factors, we noticed patients who were on thyroid replacement have a substantially lower risk of developing cancer when compared to the reference group and this needs to be externally validated. Rule‐in MT may be of further value in the study group for diagnosis and determining the extent of surgery. Surgical intervention for ITN appears to be associated with lower morbidity compared to meta‐analysis.

## Author Contributions

Study concepts: Jeyashanth Riju, Deepak Thomas Abraham, and Rekha Pai.

Study design: Jeyashanth Riju, Nihal Thomas, V. Thomas Paul, Deepak Thomas Abraham, and Rekha Pai.

Data acquisition: Jeyashanth Riju, M. Gnanasekaran, Remya Rajan, Shawn Thomas, Natarajan Ramlingam, Elanthenral Sigamani, and A. Reetu John.

Quality control of data and algorithms: Nihal Thomas, V. Thomas Paul, and Rekha Pai.

Data analysis and interpretation: Jeyashanth Riju, P. G. Maya, J. Anne Prabhu, Marie Therese, M. Paul Jacob, and C. Rajiv Michael.

Manuscript preparation: Jeyashanth Riju and Nitin Kapoor.

Manuscript editing: J. Anne Prabhu, M. Paul Jacob, J. Anish Cherian, C. Rajiv Michael, J. Amit Tirkey, H. S. Asha, and Felix Jebasingh.

Manuscript review: Nihal Thomas, V. Thomas Paul, and Deepak Thomas Abraham.

## Funding

This study was supported by Indian Council of Medical Research, under Grant No. 5/4/5‐5/Diab/22‐NCD‐II, and CMC internal fluid research grant.

## Disclosure

The manuscript has been read and approved by all the authors; the requirements for authorship as stated earlier in this document have been met; and each author believes that the manuscript represents honest work. The published work is a part of Ph.D. thesis of the Tamil Nadu Dr. M.G.R. Medical University.

## Ethics Statement

Ethics approval was obtained from Christian Medical College, Vellore. IRB min no: 13,285 dated 26/8/2020. All the procedures were done in accordance with the guidelines laid down in Declaration of Helsinki 1964 and the consent to participate was obtained prior to the procedure.

## Consent

Please see the Ethics Statement.

## Conflicts of Interest

The authors declare no conflicts of interest.

## Supporting Information

Additional supporting information can be found online in the Supporting Information section.

## Supporting information


**Supporting Information 1** Supporting–STROBE checklist.

**Supporting Information 2** Supporting table S1: Relationship of TIRADS scoring with clinic‐radiological and pathological parameters. Supporting table S2: Relationship of thyroid functional parameters and hypothyroid medication with malignancy and pathologic characteristics.

## Data Availability

The data that support the findings of this study are available on request from the corresponding author. The data are not publicly available due to privacy or ethical restrictions.
