# Supplementary material for: A Prospective Study on Challenges in the Management of Indeterminate Thyroid Nodules Without Molecular Testing
Source: Int J Endocrinol. 2026 Oct 28;2026:4705557. doi: 10.1155/ije/4705557 (PMC13717225; doi:10.1155/ije/4705557)
Supplement: Supplementary file 1 [file IJE-2026-4705557-s001.docx]

# Supplementary table S1: Relationship of TIRADS scoring with clinic-radiological and pathological parameters.

| **Variables** | **TIRADS 1-4**  **(N=207)** | **TIRADS 5**  **(N=30)** | **Unadjusted OR**  **(95% CI)** | **Univariate p value** |
| --- | --- | --- | --- | --- |
| **Age** | | | | |
| - **Median in years with IQR** | 39(IQR: 30 - 48) | 45 (IQR: 36.7 – 53.2) | 1.04(1.01-1.07) | **0.014** |
| **Gender** | | | | |
| - Male - Female | 41(19.8%)  166(80.2%) | 10(33.3%)  20(66.7%) | 2.02(0.88-4.65) | 0.097 |
| **Demography** | | |  | |
| - South India - Rest of India | 43(20.8%)  164(79.2%) | 4(13.3%)  26(86.7%) | 1.70(0.57-5.15) | 0.344 |
| **Clinical Duration** | | | | |
| **Median in months with IQR** | 12(IQR: 6 – 48) | 12(IQR 5 – 36) | 0.99(0.97-1.0) | 0.93 |
| **Thyroid related medication** | | | | |
| - No - Hypothyroid medication - Hyperthyroid Medication | 180[87%]  22[10.6%]  5[2.4%] | 26[86.7%]  4[13.3%]  0[0%] | 1.26(0.40-3.94) | 0.693 |
| **USG maximum size of nodule** | | | | |
| - Median in mm with IQR | 35(IQR 24 – 36) | 25(IQR: 15 – 45) | 0.97(0.94-0.99) | **0.03** |
| **USG Evaluation – Number of thyroid nodules** | | | | |
| **USG Evaluation –** Median number of nodules with IQR | 2(IQR: 1 – 3) | 2(IQR: 2 – 3) | 1.3(0.98-1.77) | 0.082 |
| **USG Evaluation –** Nodule component | | | | |
| - Mixed - Solid | 90[43.5%]  117[56.5%] | 6[20%]  24[80%] | 3.08(1.21 – 7.84) | **0.019** |
| **USG Evaluation –** Nodule echogenicity | | | | |
| - Hyperechoic - Hypoechoic - Isoechoic | 73[35.3%]  29[14%]  105[50.7%] | 13[43.3%]  12[40%]  5[16.7%] | 2.32(0.95-5.69)  0.27(0.9-0.78) | 0.065  **0.016** |
| **USG Evaluation –** Shape of Nodule | | | | |
| - Wider than taller - Taller than wider | 205[99%]  2[1.0%] | 27[90%]  3[10%] | 11.39(1.8-71.26) | **0.009** |
| **USG Evaluation –** Margin of Nodule | | | | |
| - Smooth - Irregular | 197[95.2%]  10[4.8%] | 26[86.7%]  4[13.3%] | 3.03(0.89-10.36) | 0.077 |
| **USG Evaluation –** Calcification | | | | |
| - No - Yes | 171[82.6%]  36[17.4%] | 8[26.7%]  22[73.3%] | 13.06(5.39-31.66) | **<0.001** |
| **FNAC Characteristics – BETHESDA Categorisation** | | | | |
| - III - IV | 178[86%]  29[14%] | 28[93.3%]  2[6.7%] | 0.44(0.10-1.94) | 0.277 |
| **TSH** (Median in mIU/L with IQR) | 1.7(IQR: 1.0-2.6) | 1.6(IQR: 0.85 – 3.02) | 0.99(0.78-1.26) | 0.927 |
| **fT4** (Median in ng/dL with IQR) | 1.12(IQR: 1.03-1.26) | 1.12(IQR: 1.05 – 1.29) | 1.55(1.12-2.15) | **0.009** |
| **Histopathology Diagnosis** | | | | |
| - Benign/NIFTP - Malignancy | 67(32.4%)  140(67.6%) | 5(16.7%)  25(83.3%) | 2.39(0.88-6.53) | 0.088 |
| **Malignancy Subtype** | | | | |
| - FvPTC - Classic PTC - Others | 90(64.3%)  42(30%)  8(5.7%) | 11(44%)  14(56%)  0(0%) | 2.73(1.14-6.51) | **0.024** |
| **Histopathology -** Advanced pathological T stage ( ≥ 3) | | | | |
| - No - Yes | 97(69.3%)  43(30.7%) | 18(72%)  7(28%) | 0.88(0.34-2.26) | 0.786 |
| Maximum size of malignant foci | 30 (IQR: 17 – 41) | 14 (IQR: 9 – 35) | 0.97(0.95-1.0) | 0.051 |
| **Histopathology -** Focality | | | | |
| - Single - Multiple | 50(35.7%)  90(64.3%) | 6(24%)  19(76%) | 0.57(0.21-1.52) | 0.259 |
| **Histopathology -** Lymphovascular Emboli | | | | |
| - No - Yes | 108(77.1%)  32(22.9%) | 17(68%)  8(32%) | 0.63(0.25-1.59) | 0.329 |
| **Histopathology -** Perineural Invasion | | | | |
| - No - Yes | 138(98.6%)  2(1.4%) | 24(96%)  1(4%) | 0.35(0.03-3.99) | 0.396 |
| **Histopathology -** Extra thyroidal extension | | | | |
| - No - Yes | 135(86.4%)  5(3.6%) | 23(92%)  2(8%) | 0.43(0.08-2.32) | 0.325 |

# Supplementary table S2: Relationship of thyroid functional parameters and hypothyroid medication with malignancy and pathologic characteristics

| Variable | TSH **≤** 1.7 mIU/L | TSH >1.7 mIU/L | p-value TSH | fT4 **≤** 1.2 ng/dL | fT4 >1.2 ng/dL | p-value fT4 | Hypothyroid medication | No hypothyroid medication | p-value medication |
| --- | --- | --- | --- | --- | --- | --- | --- | --- | --- |
| Malignancy in biopsy | 75/107 (70.1%) | 65/100 (65.0%) | 0.434 | 53/79 (67.1%) | 26/44 (59.1%) | 0.376 | 10/22 (45.5%) | 130/185 (70.3%) | **0.023** |
| Gross tumor size of dominant nodule, mm | 35.0 (26.0-50.0) | 31.0 (22.0-43.3) | **0.010** | 37.0 (25.5-48.0) | 31.5 (24.3-43.5) | 0.254 | 44.0 (26.25-49.5) | 34.0 (23.0-45.0) | 0.140 |
| Histologic subtype | FvPTC 54/75 (72.0%); Classic PTC 17/75 (22.7%); High-grade differentiated TC 2/75 (2.7%) | FvPTC 36/65 (55.4%); Classic PTC 25/65 (38.5%); Medullary thyroid Ca 2/65 (3.1%) | 0.364 | FvPTC 35/53 (66.0%); Classic PTC 16/53 (30.2%); Medullary thyroid Ca 1/53 (1.9%) | FvPTC 17/26 (65.4%); Classic PTC 8/26 (30.8%); High-grade differentiated TC 1/26 (3.8%) | 0.581 | FvPTC 4/10 (40.0%); Classic PTC 3/10 (30.0%); Medullary thyroid Ca 2/10 (20.0%); High-grade differentiated TC 1/10 (10.0%) | FvPTC 86/130 (66.2%); Classic PTC 39/130 (30.0%); High-grade differentiated TC 2/130 (1.5%); Medullary thyroid Ca 2/130 (1.5%); Tall-cell variant PTC 1/130 (0.8%) | **0.010** |
| T stage ≥ 3 | 22/75 (29.3%) | 21/65 (32.3%) | 0.717 | 21/53 (39.6%) | 4/26 (15.4%) | **0.039** | 5/10 (50.0%) | 38/130 (29.2%) | 0.284 |
| Thyroiditis - histopathology | 20/107 (18.7%) | 23/100 (23.0%) | 0.324 | 19/79 (24.1%) | 12/44 (27.3%) | 0.646 | 5/13 (38.5%) | 38/142 (26.8%) | 0.372 |
| Extrathyroidal extension - histopathology | 1/75 (1.3%) | 4/65 (6.2%) | 0.163 | 3/53 (5.7%) | 1/26 (3.8%) | 0.731 | 0/10 (0.0%) | 5/130 (3.8%) | 1.000 |
| Lymphovascular invasion | 16/75 (21.3%) | 16/65 (24.6%) | 0.645 | 12/53 (22.6%) | 4/26 (15.4%) | 0.453 | 3/10 (30.0%) | 29/130 (22.3%) | 0.579 |
| Central node metastasis | 1/75 (1.3%) | 8/65 (12.3%) | **0.029** | 3/53 (5.7%) | 3/26 (11.5%) | 0.363 | 0/10 (0.0%) | 9/130 (6.9%) | 1.000 |
| Capsular involvement | 25/75 (33.3%) | 24/65 (36.9%) | 0.679 | 19/53 (35.8%) | 8/26 (30.8%) | 0.434 | 5/8 (62.5%) | 44/77 (57.1%) | 0.771 |
| Multifocality | 49/75 (65.3%) | 41/65 (63.1%) | 0.781 | 32/53 (60.4%) | 15/26 (57.7%) | 0.819 | 9/10 (90.0%) | 81/130 (62.3%) | 0.113 |

Values are shown as n/N (%) for categorical variables and median (IQR) for continuous variables. Tumor aggressiveness variables were assessed among malignant cases where applicable.
